# Some Good Books

**Published:** 1884-02

**Authors:** 


### SOME GOOD BOOKS.

We had the pleasure, some days ago, of looking over Prof. H. C. Allen's new work on intermittent fever. It will soon be out, and is in every way a *most excellent work.* The physician who uses it will find this class of cases easy to treat hereafter. A full review will be given of it as soon as published.

Another excellent work—just out—is Dr. Minton's *Uterine Therapeutics.* A fuller notice next issue.

